# The dynamic shuttling of SIRT1 between cytoplasm and nuclei in bronchial epithelial cells by single and repeated cigarette smoke exposure

**DOI:** 10.1371/journal.pone.0193921

**Published:** 2018-03-06

**Authors:** Satoru Yanagisawa, Jonathan R. Baker, Chaitanya Vuppusetty, Takeshi Koga, Thomas Colley, Peter Fenwick, Louise E. Donnelly, Peter J. Barnes, Kazuhiro Ito

**Affiliations:** Airway Disease Section, National Heart and Lung Institute, Imperial College London, London, United Kingdom; Institute of Lung Biology and Disease (iLBD), Helmholtz Zentrum München, GERMANY

## Abstract

SIRT1 (silent information regulator 2 homolog 1) is a crucial cellular survival protein especially in oxidative stress environments, and has been thought to locate within the nuclei, but also known to shuttle between cytoplasm and nuclei in some cell types. Here, we show for the first time the dynamics of SIRT1 in the presence of single or concurrent cigarette smoke extract (CSE) exposure in human bronchial epithelial cells (HBEC). In BEAS-2B HBEC or primary HBEC, SIRT1 was localized predominantly in cytoplasm, and the CSE (3%) induced nuclear translocation of SIRT1 from cytoplasm in the presence of L-buthionine sulfoximine (an irreversible inhibitor of γ-glutamylcystein synthetase), mainly through the activation of phosphatidylinositol 3-kinase (PI3K) α subunit. This SIRT1 nuclear shuttling was associated with FOXO3a nuclear translocation and the strong induction of several anti-oxidant genes including superoxide dismutase (SOD) 2 and 3; therefore seemed to be an adaptive response. When BEAS-2B cells were pretreated with repeated exposure to a lower concentration of CSE (0.3%), the CSE-induced SIRT1 shuttling and resultant SOD2/3 mRNA induction were significantly impaired. Thus, this result offers a useful cell model to mimic the impaired anti-oxidant capacity in cigarette smoking-associated lung disease such as chronic obstructive pulmonary disease.

## Introduction

Sirtuins are nicotinamide adenine dinucleotide (NAD)-dependent protein deacetylase, which are originally found as the human homologs of silent information regulator 2 (Sir2) genes in Saccharomyces cerevisiae [1]. So far, seven isoforms of sirtuins have been identified in human (SIRT1-SIRT7) [2]. Through their different intracellular distribution [3] and specificity toward diverse acetylated substance proteins [4–6], sirtuins are known to regulate various cellular processes such as apoptosis, cellular senescence, endocrine signaling, glucose homeostasis and aging [7,8]; most of which are built on the delicate balances between oxidative vs anti-oxidative enzymes. Particularly, SIRT1 is known to deacetylate transcription factor Forkhead box O3 (FOXO3a), leading nuclear translocation of FOXO3a to stimulate transcription of anti-oxidant genes such as superoxide dismutase (SOD) [9]. Among them, SIRT1 is the most extensively studied sirtuin in mammals, and redox regulation of SIRT1 is now elucidated to be strongly associated with resolution of inflammation and cellular senescence [10–12]. In addition, the dysregulation of SIRT1 was reported in many aging associated diseases or diseases with cigarette smoke (CS)-exposure [13,14]; both of which are characterized by the aberrant oxidant / anti-oxidant regulations [15]. SIRT1 was originally found as a nuclear protein [16,17]; however recent reports have shown that SIRT1 is not anchored exclusively in the nucleus [18–20] but dynamically shuttles between the cytoplasm and nucleus [21–23]. Due to SIRT1 ability to deacetylate and modify the functions of various different substrates, such dynamic shuttling seems to be reasonable for SIRT1 to regulate cellular conditions efficiently. However, any abnormalities in SIRT1 shuttling has not investigated. Therefore, the present study was designed to clarify the dynamics of SIRT1 protein under oxidative stress and also examined the impact of cigarette smoking on SIRT1 shuttling by comparing the effects of repeated CS extract (CSE) pre-treatment with that of single CSE exposure, which might shed new light on the novel molecular mechanisms of smoking associated diseases and also serve useful *in vitro* model to mimic concurrent smoking exposure *in vivo*.

## Materials and methods

### Reagents

Commercially available reagents were obtained as follows: RPMI medium 1640 (RPMI 1640) (#11875), polymerase chain reaction (PCR) primer for SIRT1 (Hs01009005) and GNB2L1 (Hs00272002) were from Life Technologies (Carlsbad, CA, USA); fetal bovine serum (FBS), complete protease inhibitor cocktail (#11836153001), anti-SIRT6 antibody (#S4197), anti-HDAC2 antibody (#H2663), thiazolyl blue tetrazolium bromide for MTT assay (M2003), L-buthionine-sulfoximine (BSO) (#B2515), AS605240 (#A0233), rapamycin (#R8781), dimethyl biguanide hydrochloride (metformin) (#D150959), NU7026 (N1537), Z-Leu-Leu-Leu-al (MG-132) (#C2211), cycloheximide (CHX) (#C7698), resveratrol (#R5010) and sirtinol (#S7942) were from Sigma-Aldrich Co. LLC (St Louis, MA, USA); anti-SIRT1 antibody (sc-15404), PIK75 (#sc-296089), IC87114 (#sc-364509), anti-Nrf2 antibody (#sc-13032), anti-α-tubulin antibody (#sc-5286), anti-Lamin A/C antibody (#sc-7292) and anti-chromosomal region maintenance 1 (CRM1) antibody (#sc-5595) were from Santa Cruz Biotechnology (Santa Cruz Biotechnology, CA, USA); anti-β-actin antibody (#ab6276) was from Abcam plc. (Cambridge, UK); goat-derived peroxidase-conjugated anti-mouse (#P0447) or anti-rabbit (#P0448) secondary antibodies were from Dako (Cambridge shire, UK); anti-phospho-SIRT1 (Ser47) antibody (#2314), anti-phospho-Akt (Ser47) (p-Akt) antibody (#4060) and anti-total Akt antibody (#4691) were from Cell Signaling Technology (Danvers, MA, USA); GSK2636771 (#S8002), BIRB796 (#S1574) and U1026 (#S1102) were from Selleck biochemicals (Houston, TX, USA); N-acetyl-Leu-Leu-Norleu-al (ALLN) (#208750) and Akt inhibitor (Akt Inhibitor II) were from Millipore (U.K.) Limited (Hertfordshire, U.K.).

### Cells

The BEAS-2B cell line (SV40-immortalized human airway bronchial epithelial cell line) were purchased from the American Culture of Tissue Collection, and were grown in complete growth medium (RPMI 1640 supplemented with heat-inactivated 10% FBS and 1% L-glutamine) at 37 °C / 5% CO_2_. Before use, cells were starved for 24 hours in minimum medium (RPMI 1640 supplemented with 1% FBS and 1% L-glutamine). In the BSO pretreatment model, cells were pre-incubated with BSO for 16–18 hours. Various inhibitors or activators were added 30 min prior to the cigarette smoke extract (CSE) exposure. Primary cells were obtained as follows: human airway epithelial cells of bronchial origin (hAEC) and air-liquid interface (ALI) cultured human bronchial epithelial cells (HBEC) were from Epithelix-Sarl (Geneva, Swiss); human pulmonary artery endothelial cells (hPAEC) were from Lonza (#CC-2530, Slough, UK). For the analysis of clinical samples, human primary bronchial epithelial cells were extracted from lung tissue from patients with chronic obstructive pulmonary disease (COPD) undergoing lung resection surgery at the Royal Brompton Hospital. The characteristics of patients with chronic obstructive pulmonary disease (COPD) were as follows (mean ± SD); 3 male and 1 female, 68.75 ± 9.8 years old, pack year (number of cigarettes smoked per day/20 x duration of smoking): 82.0 ± 58.7, FEV_1_ (forced expiratory volume in one second): 1.56 ± 3.1L (62.0 ± 18.2% predicted normal), FEV_1_/FVC (forced vital capacity): 49.0 ± 14.0%. All subjects gave informed written consent and the study was approved by the NRES London-Chelsea Research Ethics committee (study number 09/H0801/85). The cells were cultured as monolayers in LHC-9 media (Invitrogen, Paisley, UK) on collagen (1% w/v) coated plates.

### Preparation of CSE

One full-strength Marlboro cigarette with filter removed (Phillip Morris, London, UK) was bubbled into 10 ml of minimum medium, at a rate of one cigarette per 1.5 minutes. CSE was then passed through a 0.2μm filter to sterilize and remove particulate matter and was used immediately. The optical density was measured at 320λ wavelength, and the solution was diluted to be OD = 0.85 (this is original stock as 100%). The stock CSE was thereafter diluted with culture media to appropriate percentages of CSE solution.

### Protein extraction and Western blotting (WB)

**Fig 1:**
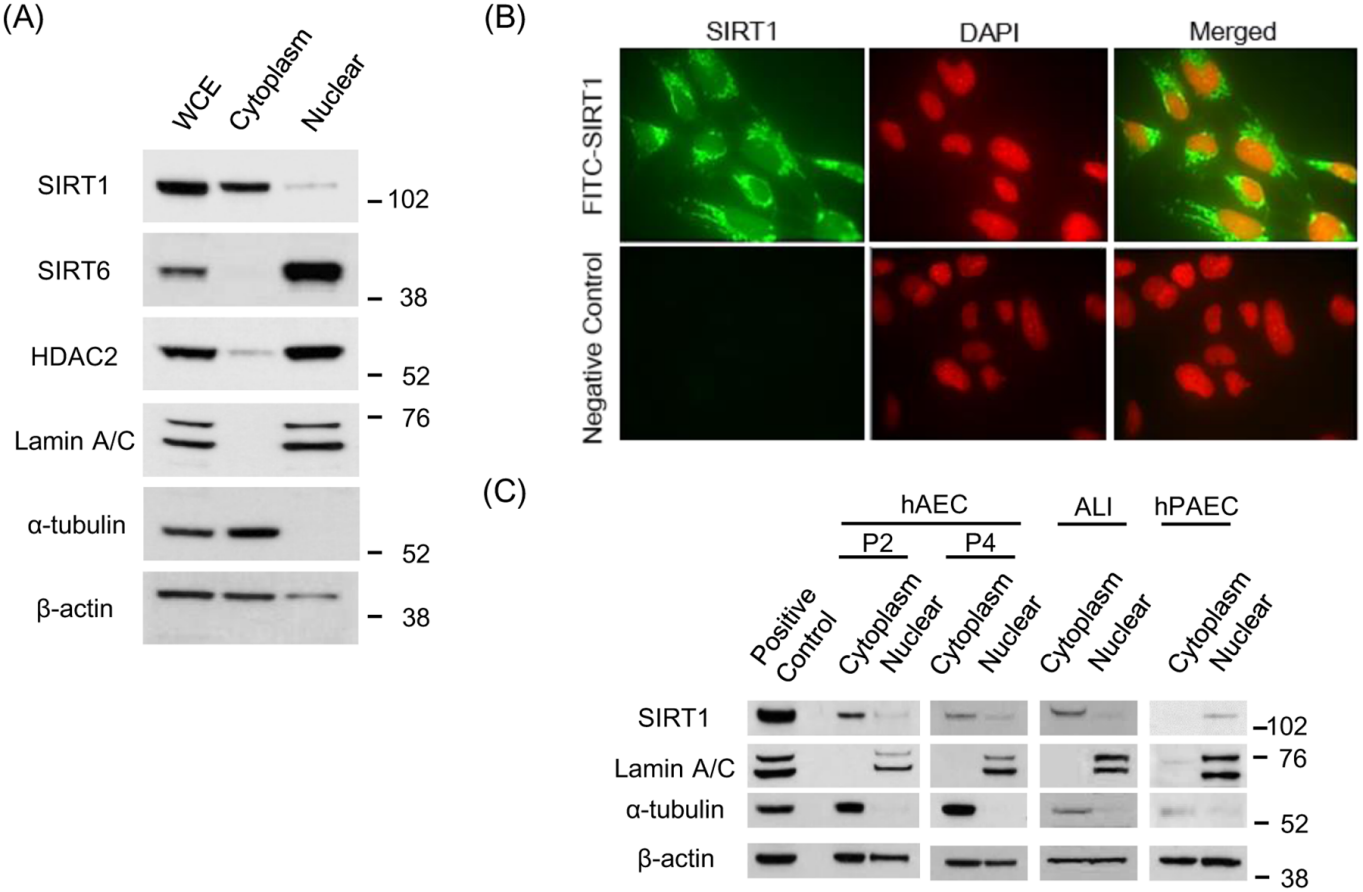
Localization of SIRT1 in human bronchial epithelial cells. (A) SIRT1, SIRT6 and HDAC2 protein expressions are shown in cytoplasmic and nuclei fraction. Lamin A/C and α-tubulin was used for loading control of nuclear and cytoplasmic fractions, respectively. (B) SIRT1 localization in BEAS-2B cells was detected by immunocytochemistry. Upper panel showed SIRT1 immunoreactivity, and lower panel indicated negative control (× 40 in BD Pathway 435 bioimager). (C) SIRT1 subcellular localization was examined for the different human primary cells, such as human airway epithelial cells of bronchial origin (hAEC, passage 2 and 4), air-liquid interface cultured human bronchial epithelial cells (ALI) and human pulmonary artery endothelial cells (hPAEC).

**Fig 2:**
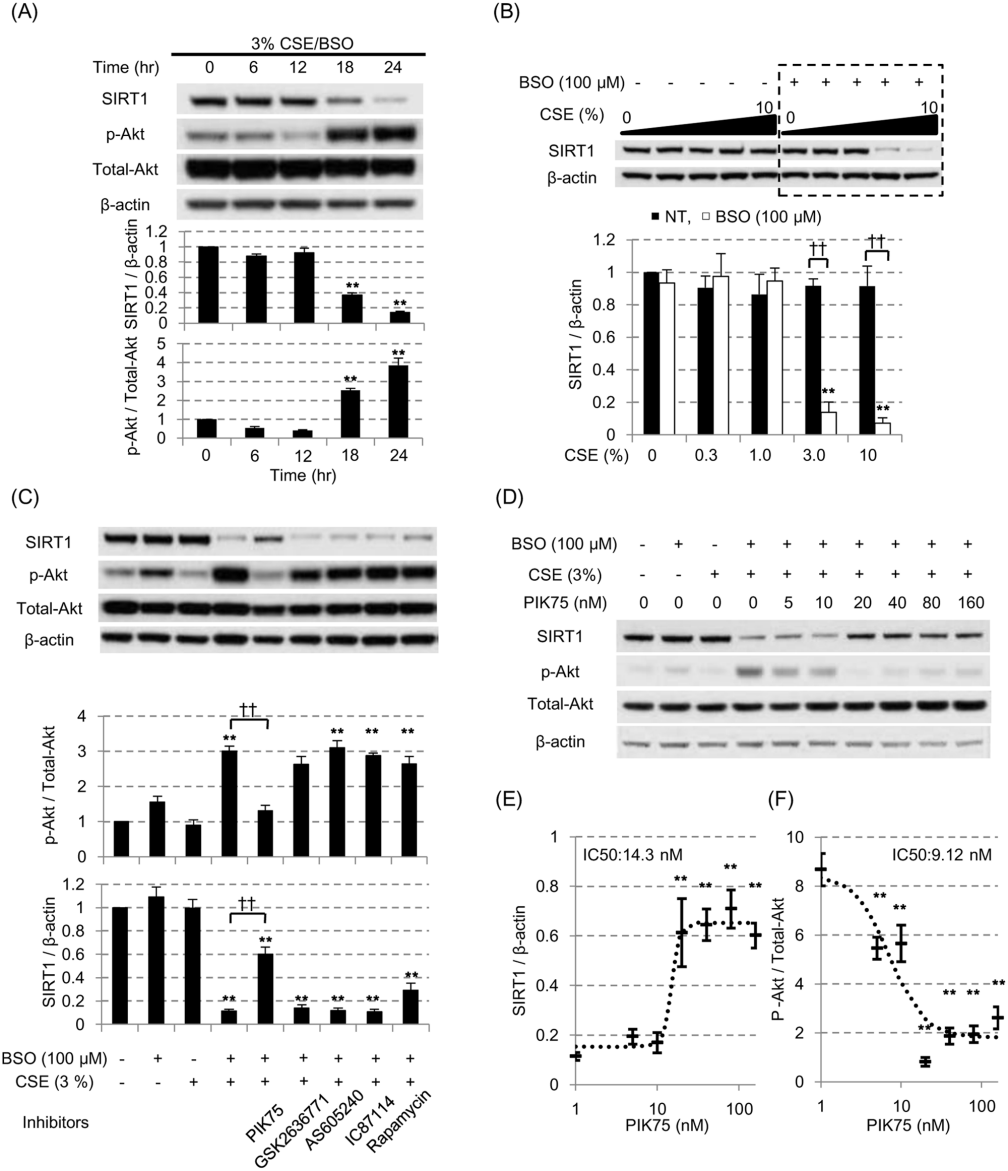
PI3Kα is involved in the CSE-induced SIRT1 reduction in the presence of L-buthionine-sulfoximine (BSO) in cytoplasmic fraction. (A) After pretreatment with 100 μM BSO for 16 hours, BEAS-2B cells were exposed to the 3% CSE for up to 24 hours. SIRT1 protein and phosphorylated Akt/Akt ratio(p-Akt / Total-Akt) were assayed at different time point by SDS-PAGE / WB in cytoplasmic fraction. (B) SIRT1 protein levels were examined at different concentration of CSE with or without 100 μM BSO pretreatment. All values were mean value ± SEM of at least three experiments. ** *p* < 0.01, compared with the values of non-treatment group; ^††^ *p* < 0.01 between the absence or presence of BSO. (C) Various inhibitors against PI3K signaling pathway molecules were added 30 min prior to the CSE exposure (0.1 μM of PIK75 for PI3Kα inhibitor, 10 μM of GSK2636771 for PI3Kβ inhibitor, 10 μM of AS605240 for PI3Kγ inhibitor, 5 μM of IC87114 for PI3Kδ inhibitor, and 0.02 μM of rapamycin for mTOR inhibitor). The Akt activation status (p-Akt / Total-Akt) and SIRT1 protein levels were evaluated by SDS-PAGE / WB. (D, E) The concentration dependent effects of PIK75 on the SIRT1 and Akt phosphorylation levels were examined using CSE exposure with BSO pretreatment model. The ratio of p-AkT/Total-Akt or SIRT1 levels were fitted with the sigmoid-curve, and calculated for the IC_50_ of PIK75 (E, respectively). All values were mean value ± SEM of at least three experiments. ** *p* < 0.01, compared with the values of non-treatment group; ^††^ *p* < 0.01 between the two groups.

Cytoplasmic fraction was prepared using modified radio-immuno-precipitation assay (RIPA) buffer (50 mM Tris HCl pH 7.4, 0.5% NP-40, 0.5% w / v Na-deoxycholate, and 150 mM NaCl) with freshly added complete protease inhibitor cocktail. This lysis buffer was confirmed to extract cytoplasmic rich fraction based on β-actin and Lamin A/C staining in Western blotting. This method has been used for the experiments shown in Figs 1 and 2, S1 and S2 Figs. In following experiments, cytoplasmic fraction and nuclear fraction were prepared using Nuclear Extract Kit (#40010, Active Motif, La Hulpe, Belgium) according to the manufacture’s instruction.

The cell extracts prepared above were separated by sodium dodecyl sulfate-polyacrylamide gel electrophoresis (SDS-PAGE), transferred to nitrocellulose membrane, and then incubated overnight with anti-SIRT1 antibody (1:1,000 dilutions, #sc-15404), anti-SIRT6 antibody (1:3,000 dilutions), anti-HDAC2 antibody (1:200,000 dilutions), anti-p-Akt antibody (1:500 dilution), anti-Total-Akt antibody (1:1,000 dilutions), anti-Nrf2 antibody (1:1000, dilutions) or anti-CRM1 antibody (1:200 dilutions). To standardize the expression of each protein, the membranes were re-probed with anti-β-actin antibody (1:200,000 dilutions) or anti-α-tubulin antibody (1:2,000 dilutions), anti-Lamin A/C antibody (1:500 dilutions). The membranes were then incubated with the appropriate peroxidase-conjugated secondary antibodies (1:3000 dilutions each). The bound antibodies were visualized by chemiluminescence (ECL plus; GE healthcare, Buckinghamshire, UK).

### MTT assay

After the removal of supernatant, cells were washed with phosphate buffered saline (PBS) once, and incubated with 1 mg / ml thiazolyl blue tetrazolium bromide solution for 1 hour at 37 °C / 5% CO_2_. Formazan crystals were dissolved in DMSO, and optical density was measured at 550 λ wavelength. Cell viabilities were represented as the relative ratio to that of non-treatment group.

### Protein half-life assay

After 16 hours pretreatment with or without 100 μM BSO, BEAS-2B cells were exposed with or without 3% CSE from 1 to 24 hours in the presence of 30 min pre-incubation with cycloheximide (CHX) (100 ng / ml). At different time points, cells were harvested and SIRT1 protein levels were assayed by SDS-PAGE / WB.

### Reverse transcriptase quantitative PCR (RT-qPCR)

Total cellular RNA was extracted using RNeasy mini kit (Qiagen, Valencia, CA, USA) and cDNA was prepared by using Multiscribe reverse transcriptase (Applied Biosystems, Warrington, UK). The RT-qPCR analysis of SIRT1 and GNB2L as a house keeping gene, were performed using Taqman primers and probe set from Applied Biosystems in a Corbett Rotor-Gene 3000 (Corbett Research Sortlake, NSW, Australia).

### Analysis of nascent protein synthesis

Nascent protein synthesis was measured by modifying the protocol for Click-IT^®^ L-homopropargylglycine (HPG) Alexa Fluor Protein Synthesis Assay kits (#C10429, ThermoFisher scientific, Rockford, IL, USA). In brief, cells were at first cultured with methionine free RPMI medium (#14517–01, ThermoFisher scientific) for 30 min. Then medium was replaced with CSE- or vehicle-containing methionine free RPMI supplemented with 50 μM Click-IT^®^ HPG, which was incorporate into the newly synthesized proteins instead of methionine. After 6 hours, cells were harvested and lysed for WCEs preparation. The WCEs products were immuno-precipitated by rabbit-derived anti-SIRT1 antibody (#sc-15404, Santa Cruz Biotechnology) with the Protein A magnetic beads (#10001D, Life Technologies), and additionally labelled with Biotin-Azide (#B10184, ThermoFisher scientific) in the presence of cupper, according to the manufacture’s instruction. After separation with SDS-PAGE / WB, biotin labelled SIRT1 (biotin-SIRT1) was then detected by the HRP-conjugated streptavidin (1:1,000 dilutions, ThermoFisher scientific). To standardize the biotin-SIRT1, the membranes were re-probed with mouse-derived anti-SIRT1 antibody (1:8,000 dilutions, #SAB1404972, Sigma-Aldrich), and then incubated with the goat-derived anti-mouse peroxidase-conjugated secondary antibodies (1:3000 dilutions). Newly synthesized SIRT1 was expressed as relative intensity calculated by the ratio of the mean band intensity for biotinylated SIRT1 to the mean band intensity for the total SIRT1 protein.

### SIRT1 motif change by oxidative stress

The various antibodies against different part of SIRT1 were used for examining the protein motif changes caused by oxidative stress. We used anti-SIRT1 antibodies from; Santa-cruz technology (#sc-15404 for ① indicated in Panel F in S2 Fig. (F)), Sigma-Aldlich (#5322 for ②, #5447 for ③, #SAB1404972 for ④); Cell signaling technology (#9475 for ⑤); and OriGene (Rockville, MD, USA) (#TA303784 for ⑥).

### Immunofluorostaining for SIRT1 in BEAS-2B cells

Immunostaining of SIRT1 was done using Histostain-*Plus*^®^ IHC kit (#85–9043, Invitrogen, Paisely, UK). Briefly, BEAS-2B cells were cultured on the chamber slide, and then fixed in 3% formaldehyde fixative solution for 15 min, and peameabilized by 0.1% triton X-100 in 10 mM PBS for 30 min. After blocking with serum blocking solution (#85–9043, Invitrogen), the cells were incubated with FITC-conjugated anti-SIRT1 polyclonal antibody (1:100 dilution, #orb103489, biorbyte, Cambridge, UK) overnight at 4 °C. The immunoreactions were then embedded in the DAPI (4’,6-diamidino-2-phenylindole) containing mounting solution (ProLong^®^ Diamond Antifade Mountant with DAPI, ThermoFisher scientific, Rockford, IL, USA), as manufacture’s instruction. The slides were analyzed by BD Pathway 435 bioimager system (with 40X Olympus Plan Fluorite objective, 0.75 numerical aperture, Lumenera-Infinity 3–1 12 bit CCD camera) at room temperature using BD Atto Vision softeware (BD, New Jersey, US).

### Malondialdehyde (MDA) assay

MDA was quantified using OxiSelect^™^ TBARTS assay kit (#STA-330, Cambridge Bioscience, Cambridge, UK). Briefly, the cytoplasm samples were thawed on ice and centrifuged for 1 or 2 min at 4°C, 13000rpm, before use. Twenty μl of diluted samples (0.5μg/mL protein) or standards were mixed and incubated with equal volume of SDS lysis solution for 5min at room temperature (RT) with shaking in PCR plates. TBA reagent was then added and mixed with the samples and after 5–15 min at RT was set up for 1 hour at 100°C in a PCR cycler. The plates were then incubated for 15 min at 4°C in a refrigerator. Fifty μl of this solution was then transferred to a half- area 96-well black plate and MDA was detected by fluorescence with 540λ excitation and 590λ emission. The amount of MDA in the samples was calculated using the standard curve.

### Statistical analysis

The *in vitro* data using BEAS-2B cells were expressed as mean values ± SEM. Data were analyzed by one way ANOVA followed by Turkey’s or Sheffe’s F-test to adjust for multiple comparisons (Statcel 2, OMS publishing Inc., Saitama, Japan). An unpaired two-tailed Student’s t-test was used for single comparisons. For the analysis of clinical samples, data were expressed as mean values ± SD, and statistical significance was assessed by Mann-Whitney U test for single comparisons, or by using non-parametric Shirley-Williams test. All reported *P* values are two-sided, and *P* values of less than 0.05 were considered statistically significant.

## Results

### SIRT1 is localized in cytoplasm in human bronchial epithelial cells

SIRT1 has traditionally been reported to locate mainly within the nucleus, but some reports elucidated that the SIRT1 potentially exists either in the nucleus or cytoplasm, as SIRT1 exerts different function with diverse substrate at each subcellular component. As shown in Fig 1A, SIRT1 is localized in cytoplasm of BEAS-2B cell line at normal culture condition, which was obviously contrast to the other de-acetylating enzymes SIRT6 and HDAC2; both of which located exclusively in the nucleus (Fig 1A). Immunocytochemistry experiment also confirmed abundant SIRT1 located mainly within the cytoplasm (Fig 1B). As BEAS-2B cell line is an immortalized bronchial epithelial cell line, we also investigated the localization of SIRT1 in human primary airway epithelial cells of bronchial origin (hAEC: passage 2 and 4) in monolayer culture and air-liquid interface (ALI)-cultured human bronchial epithelial cell (HBEC). In both type of bronchial cells, SIRT1 was located mainly within cytoplasm (Fig 1C). On the other hand, SIRT1 was found within the nucleus in human pulmonary artery endothelial cells (hPAEC), suggesting that the distribution of SIRT1 differed from one cell type to another as previously reported [22,24].

### Cigarette smoke extracts decreased cytoplasmic SIRT1

As shown in Panel A and B in S1 Fig, CSE exposure caused a reduction in SIRT1 protein expression in cytoplasmic fractions in a concentration and time-dependent manner. SIRT1 protein expression was significantly decreased at 20% CSE although the cell viability was significantly reduced at higher than 10% CSE (61 ± 12% reduction).

Therefore, cells were treated with L-buthionine sulfoximine (BSO: an irreversible inhibitor of γ-glutamylcystein synthetase [25]), which depletes intracellular glutathione storage, making cells more susceptible to the exogenous oxidative stress [25] without increasing cell toxicity. In fact, it has been reported that available glutathione storage is decreased in the chronic smokers [26,27]. As shown in Fig 2A and 2B, in the presence of BSO, cytoplasmic SIRT1 was decreased by lower concentrations of CSE (3%) after 24 hours exposure. No significant reduction of cell viability was seen.

At same time, the 3% CSE exposure with the pretreatment of 100 μM BSO (3% CSE/BSO) significantly increased the phosphorylation of Akt (Fig 2A), suggesting the phosphatidylinositol 3-kinase (PI3K) signaling pathway was activated in this condition. To identify the isoform of PI3K involved in SIRT1 reduction, we tested various inhibitors of PI3K pathway (0.1 μM of PIK75 for PI3Kα inhibitor, 10 μM of GSK2636771 for PI3Kβ inhibitor, 10 μM of AS605240 for PI3Kγ inhibitor, 5 μM of IC87114 for PI3Kδ inhibitor, and 0.02 μM of rapamycin for mTOR inhibitor). As shown in Fig 2C, only PIK75 among all inhibitors tested suppressed the CSE-induced Akt phosphorylation effectively (Fig 2C, upper panel), and significantly reversed the reduction of SIRT1 protein under oxidative stress (Fig 2C, lower panel). PIK75 concentration-dependently reversed SIRT1 suppression (Fig 2D and 2E) with an IC_50_ value of 14.3 nM and suppressed p-Akt/Total-Akt ratio (Fig 2E and 2F) with an IC_50_ value of 9.1 nM, respectively; these values were very close to the previously reported IC_50_ of PIK75 for PI3Kα enzyme activity (IC_50_: 5.8 nM) [28]. Therefore, these results suggested that PI3K, especially α subunit, plays an important role in regulating SIRT1 suppression under oxidative stress. Furthermore, we also examined the effect of NU7026 (DNA dependent protein kinase (DNA-PK) inhibitor, 3.3 μM) as PIK75 is also known to inhibit DNA-PK [28], and also tested other signaling inhibitors such as 1,1-dimethyl-biguanide hydrochloride (5’ AMP-activated protein kinase (AMPK) activator, 5 mM), BIRB796 (p38 mitogen activated protein kinase (MAPK) inhibitor, 1 μM) and U1026 (MAPK / extracellular-signal-regulated kinases (ERK) kinase (MEK) 1 / 2 inhibitor, 1 μM), however none of which had any effects on SIRT1 suppression.

To clarify the mechanism of reduction of cytoplasmic SIRT1, we investigated role of proteasome degradation using proteasome inhibitors, Z-Leu-Leu-Leu-al (MG-132: 8 μM) or N-Acethyl-Leu-Leu-Norleu-al (ALLN: 10 μM) (Panel A in S2 Fig), SIRT1 protein half-life in the presence of cycloheximide (Panel B in S2 Fig) and the translational regulation of SIRT1 protein using Click-IT^®^ assay (Panel C in S2 Fig), but none of systems were responsible for the reduction of cytoplasmic SIRT1. We also investigated the level of SIRT1 gene transcription determined by SIRT1 mRNA levels by reverse transcriptase quantitative polymerase chain reaction (RT-qPCR). Although the 24 hours exposure with CSE decreased the SIRT1 mRNA only in the presence of BSO (Panel D in S2 Fig), the reduction was not reversed by PIK75 (Panel E in S2 Fig). Additionally, we had concerns on the modification of the antibody recognition motif of SIRT1 by oxidative stress, resulting in failure of SIRT1 detection by the antibody used rather than actual reduction of SIRT1 protein. Therefore, we tested various antibodies that recognize epitopes within different regions of the SIRT1 protein, however, all antibodies confirmed the reduction of SIRT1 protein levels by CSE exposure with BSO pretreatment (Panel F in S2 Fig), therefore antibody detection motif on SIRT1 was not modified by oxidative stress. Finally, to investigate the possibility of secretion of SIRT1 as the mechanism of cellular SIRT1 reduction, we also detected SIRT1 in the cell culture supernatant, but no significant increase in SIRT1 protein after CSE exposure was observed (Panel G in S2 Fig).

### Cigarette smoke extracts induced shuttling of SIRT1 from cytoplasmic to nuclei

**Fig 3:**
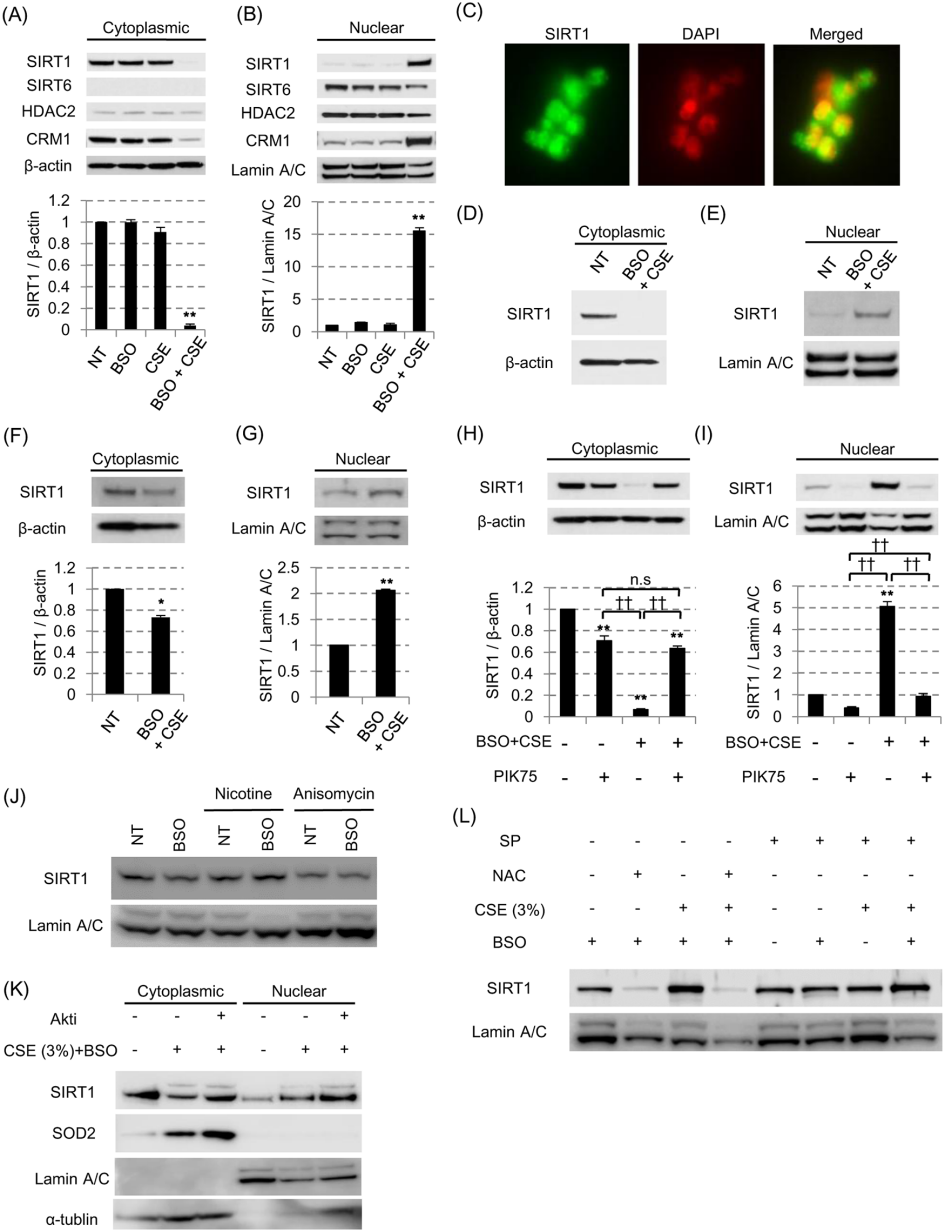
CSE-induced SIRT1 reduction is actually due to shuttling of cytoplasmic SIRT1 to nuclei. BEAS-2B cells were pretreated with or without 100 μM BSO in the presence or absence of 3% CSE. After 24 hours, localization of SIRT1, SIRT6, HDAC2 and CRM1 in cytoplasm (A) and nuclei (B) were examined by SDS-PAGE / WB. (C) SIRT1 localization after CSE exposure for 24 hours in the presence of BSO in BEAS-2B cells were detected by immunocytochemistry (× 40 in BD Pathway 435 bioimager). (D-G) In the 3% CSE exposure with 100 μM BSO pretreatment model, the SIRT1 shuttling was examined in the human primary bronchial epithelial cells (D, E) or air-liquid interface cultured human bronchial epithelial cells (F, G). (H, I) After CSE exposure with BSO pretreatment in BEAS2B, the effect of PIK75 (0.1 μM) on the SIRT1 shuttling was examined. (J) SIRT1 in nuclear fraction after nicotine (10 μM) or anisomycin (10 μg / ml) treatment for 6 hours in BEAS-2B. (K) Effects of Akt inhibitor (10 μM) on SIRT1 protein in nuclei from cells stimulated with 3% CSE for 24 hours. (L) Effects of N-acetyl cysteine (NAC, 10 mM) and SP-600125 (SP, 10 μM) on SIRT1 protein in nuclei from cells stimulated with 3% CSE for 24 hours. All values were mean value ± SEM of at least three experiments. * *p* < 0.05, ** *p* < 0.01, compared with the values of non-treatment group; ^††^ *p* < 0.01 between the two groups.

As shown above, none of the mechanisms such as transcriptional, translational, or post-translational regulations, seemed to be associated with SIRT1 reduction in cytoplasmic fraction under oxidative stress. Therefore, we next investigated the localization of SIRT1 under oxidative stress. Interestingly, cytoplasmic SIRT1 dramatically translocated to nucleus after 24 hour exposure of CSE in the presence of BSO in BEAS-2B cells (Fig 3A and 3B, S3 Fig, Panel A in S4 Fig). This dynamic shuttling between cytoplasm and nucleus seemed to be SIRT1 specific phenomenon, because both SIRT6 and HDAC2 stayed within the nucleus irrespective of the CSE stimulation with BSO pretreatment; in case of SIRT6 or HDAC2, only nuclear protein fractions had a tendency of reduction under oxidative stress without changes in its location. Immunocytochemistry also showed nuclear localization after CSE treatment (Fig 3C) compared to the baseline in Fig 1B. Most importantly, the shuttling of SIRT1 to nuclei by CSE was also confirmed in primary cells, such as hAEC or ALI-cultured HBEC (Fig 3D and 3E or 3F and 3G, respectively). The shuttling of SIRT1 seemed to be caused by the active transport because its dynamics was in parallel to that of chromosome maintenance region 1 (CRM1) protein (Fig 3A and 3B), which is known to recognize nuclear export sequence and function as a cargo for SIRT1 nuclear export [22]. Furthermore, the nuclear translocation of SIRT1 under oxidative stress in BEAS-2B cells was almost completely reversed by PIK75, the PI3Kα specific inhibitor (Fig 3H and 3I). Thus, the SIRT1 located mainly in cytoplasm in bronchial epithelial cells at normal condition, and translocated from cytoplasm to nucleus under oxidative stress, which was mediated mainly through PI3Kα signaling. The SIRT1 shuttling was not induced by nicotine, an Akt activator [29] as shown in Fig 3J; in addition, Akt inhibitor did not inhibit 3% CSE-induced SIRT1 nuclear translocation, either (Fig 3K). This suggests that the translocation of SIRT1 is regulated downstream of PI3Kα before Akt comes into play. The SIRT1 shuttling was not induced by anisomycin, a JNK activator at 6 hours or 24 hours after treatment (Fig 3J). SP-600125, a JNK inhibitor, did not inhibit CSE-induced SIRT1 shuttling, either (right panel in Fig 3L). Although Nasrin and colleagues reported that JNK is involved in SIRT1 shuttling to the nuclei in hydrogen peroxide stimulated human embryonic kidney (293T) cells [30], it was not the case in our current study with cigarette smoke conditioned media. At least, N-acetyl cysteine clearly inhibited CSE-induced SIRT1 shuttling, suggesting oxidative stress to drive the shuttling process (left panel in Fig 3L).

**Fig 4:**
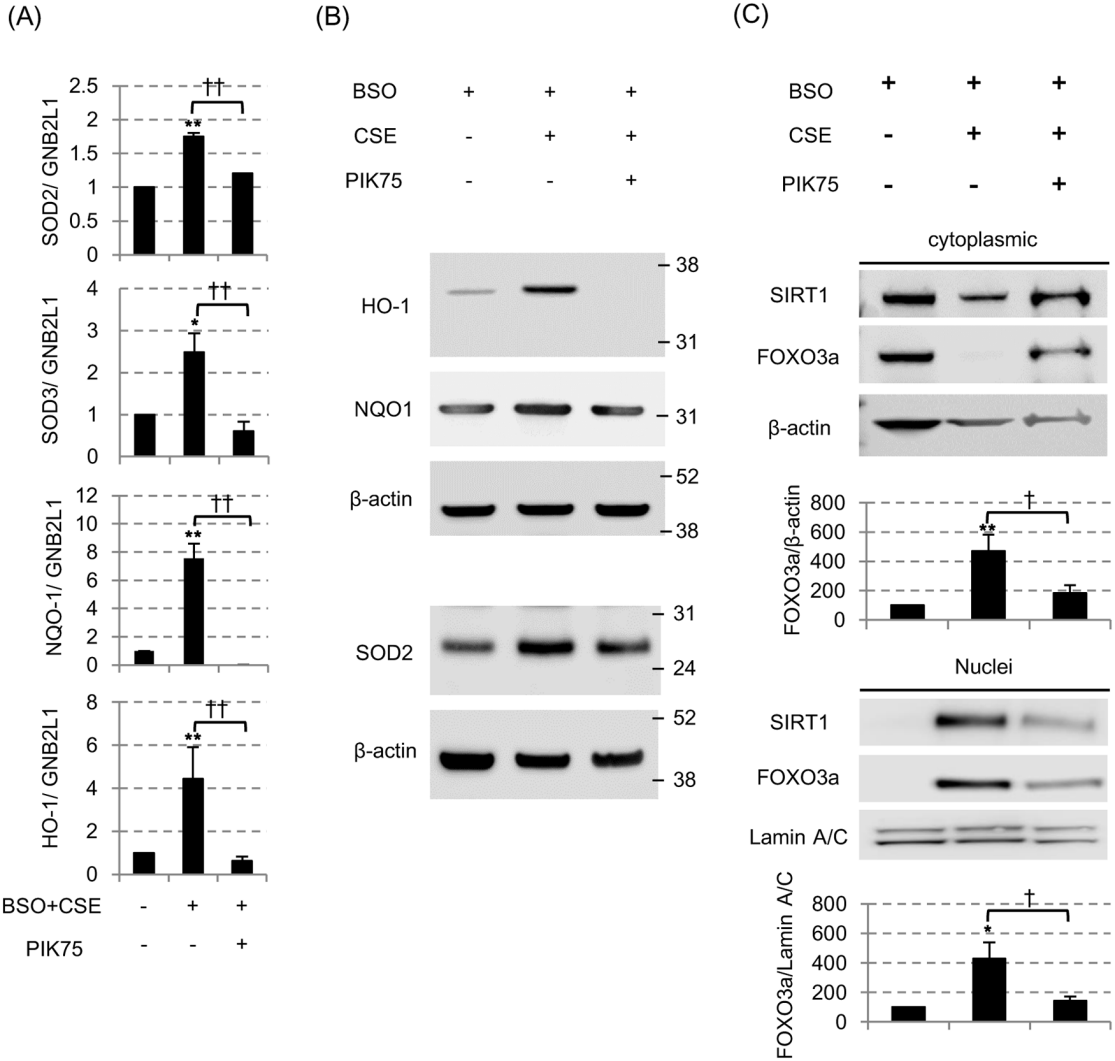
CSE-induced SIRT1 nuclear translocation was associated with increases in anti-oxidant gene and protein expression. (A) The messenger RNA levels of superoxide dismutase 2 (SOD2), SOD3, NADPH quinone oxidoreductase-1 (NQO-1) and hemoxygenase-1 (HO-1), all of which were normalized to mRNA expression of a GNB2L1 housekeeping gene, were assessed by the reverse transcription-quantitative polymerase chain reaction (RT-qPCR) 24 hours after 3% CSE exposure with BSO. All values were mean values ± SEM of at least three experiments. * *p* < 0.05, ** *p* < 0.01, compared with the values of non-treatment group; ^††^ *p* < 0.01, compared between the two groups. (B) The protein levels of HO-1, NQO-1 and SOD2 were assessed by SDS-PAGE/WB. (C) SIRT1 and FOXO3a protein in cytoplasmic fraction (top) and nuclear fraction (bottom) 24 hours after 3% CSE exposure with BSO. PIK75 (0.1 μM) was also treated before CSE exposure. The band density of SIRT1 and FOXO3a were also calculated and corrected to that of Lamin A/C. * *p* < 0.05, ** *p* < 0.01, compared with the values of BSO only group; ^†^ *p* < 0.05, compared between with or without PIK75.

The shuttling of cytoplasmic SIRT1 to the nucleus seemed to be cell-protective, because nuclear SIRT1 is strongly associated with the mRNA induction of some anti-oxidative proteins; not only with the well-known SIRT1 downstream target superoxide dismutase 2 (SOD2) [31], but also with the hemoxygenase-1 (HO-1) (Fig 4A), SOD3 (Fig 4B) and NADPH quinone oxidoreductase-1 (NQO-1). Such beneficial effect (mRNA induction of anti-oxidative proteins) was also confirmed to be inhibited by the PI3Kα inhibitor, PIK75 (Fig 4A). As well as in mRNA levels, HO-1 and SOD2 proteins were also induced by CSE and inhibited by PIK-75 (Fig 4B). Furthermore, although FOXO3a is known to be an important transcription factor to induce these anti-oxidant gene expression and also the function is regulated by SIRT1 [32], we found 3% CSE also increased FOXO3a in nuclei together with SIRT1 (Fig 4C). Taken together, SIRT1 translocation from cytoplasm to nucleus is a normal protective mechanism against “single” oxidative stress, by induction of potent anti-oxidant enzymes.

### Repeated low concentrations cigarette smoke exposure impaired the SIRT1 shuttling to nuclei

**Fig 5:**
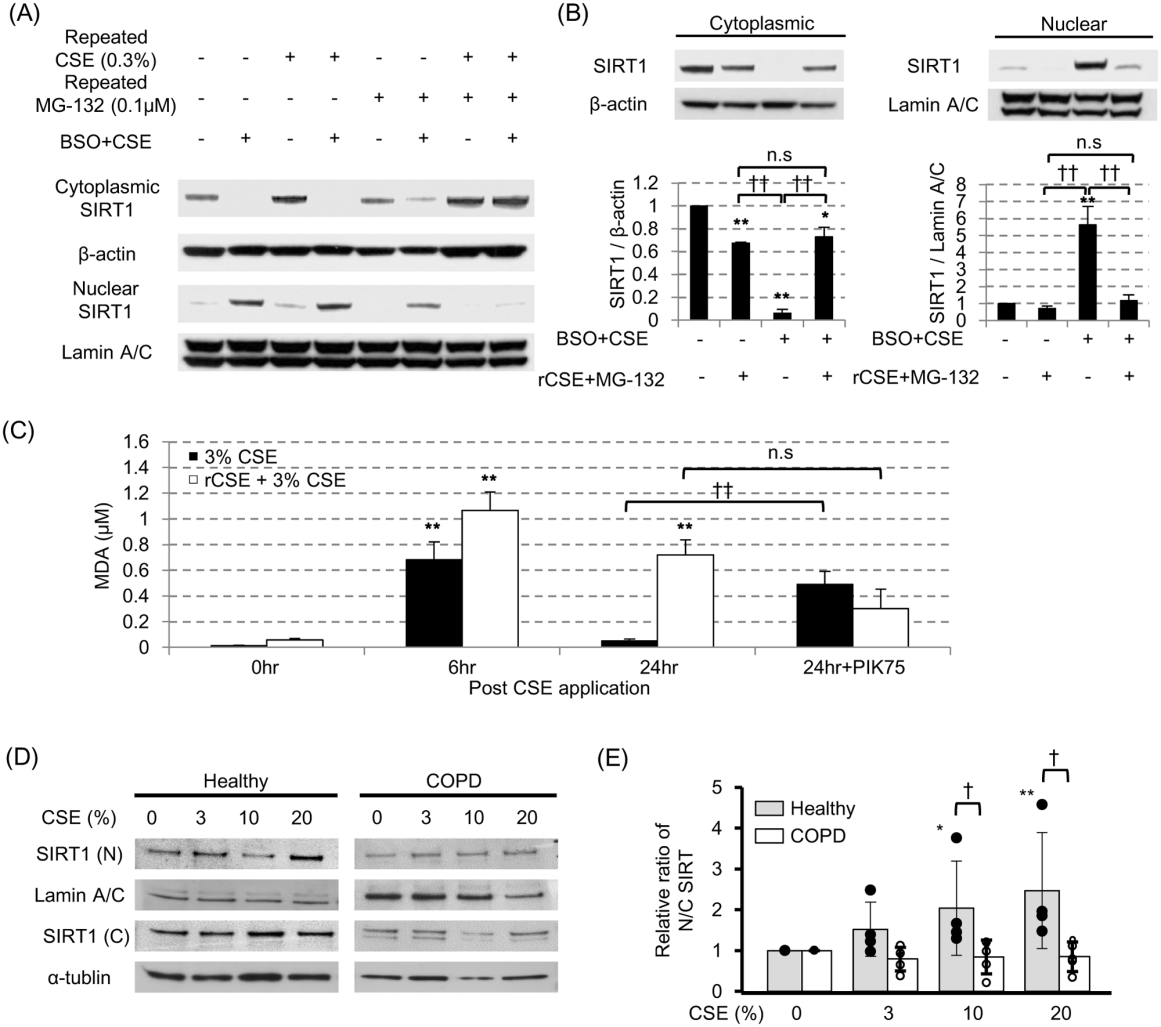
Shuttling of SIRT1 to nuclei was impaired by repeated CSE priming and in COPD. (A) In CSE exposure with BSO pretreatment model, the effects of different concentration of recurrent CSE in the presence or absence of repeated 0.1 μM MG-132 were examined. (B) The effect of 0.3% CSE in the presence of 0.1 μM MG-132 on SIRT1 shuttling under the oxidative stress was confirmed. (C) Cellular oxidative stress determined as malondialdehyde in 3% CSE stimulated and in repeated CSE priming with 3% CSE stimulated cells. (D) SIRT1 proteins in nuclei [N] and cytoplasm [C] in CSE exposed primary bronchial epithelial cells from subjects with or without COPD. (E) Nuclear to cytoplasmic (N/C) ratio of SIRT1 in experiments (D). The data were shown as the relative ratio of that of non-treatment group. * *p* < 0.05, ** *p* < 0.01, compared with the value of non-treatment group; ^†^ *p* < 0.05, ^††^ *p* < 0.01, compared between the two group.

SIRT1 nuclear shuttling seems to be important adaptive response against oxidative stress. However, the protective mechanism (such as anti-oxidant capacity) is known to be impaired in some diseases, especially chronic obstructive pulmonary disease (COPD). As the chronic exposure of cigarette smoke or indoor smoke is the major risk factor for the pathogenesis of COPD, we set up a cell model primed by the repeated cigarette smoke exposure. As shown in Fig 5A and 5B (and Panel B in S4 Fig), pre-treatment of BEAS-2B cells with a lower concentration of CSE (0.3%, every 24 hours, x3) in combination with MG-132 (0.1 μM, every 24 hours, x3, to avoid any effects of proteasome degradation of SIRT1 or other proteins) almost completely inhibited SIRT1 nuclear translocation by CSE (3%) exposure in the presence of BSO (100 μM). This suggested that the repeated exposure of low concentrations of CSE impaired the protective mechanism against oxidative stress by inhibition of SIRT1 shuttling. As this impairment of SIRT1 shuttling was not observed by repeated CSE without MG-132 (Fig 5A), degradation of SIRT1 or any other proteins will affect the outcome seen in Fig 5B. In addition, we determined cellular oxidative level as malondialdehyde (MDA). As shown in Fig 5C, single treatment of 3% CSE increased oxidative stress at 6 hours after stimulation, but disappeared at 24 hours, although this was reversed by PI3Kα inhibitor PIK75, suggesting induction of anti-oxidants are involved in reduced oxidative stress at 24hours. In contrast, when cells were primed with repeated low concentration CSE, the level of oxidative stress was still high at 24 hours after CSE stimulation. Thus, impairment of SIRT1 nuclear shuttling causes exposure of higher level of oxidative stress continuously due to no induction of anti-oxidants.

We also examined the SIRT1 shuttling of primary bronchial epithelial cells obtained from the healthy subjects (n = 4) or COPD patients (n = 4). In normal subjects, the nuclear to cytoplasmic (N/C) ratio of SIRT1 were CSE dose-dependently increased, however SIRT1 nuclear shuttling seemed to be impaired in those from COPD subjects (Fig 5D and 5E), which were significant compared with the healthy subjects at higher than 10% CSE (Fig 5D and 5E). These results suggested the impaired SIRT1 shuttling in the patients with COPD, which were compatible with our recurrent CSE exposure priming models *in vitro*.

**Fig 6:**
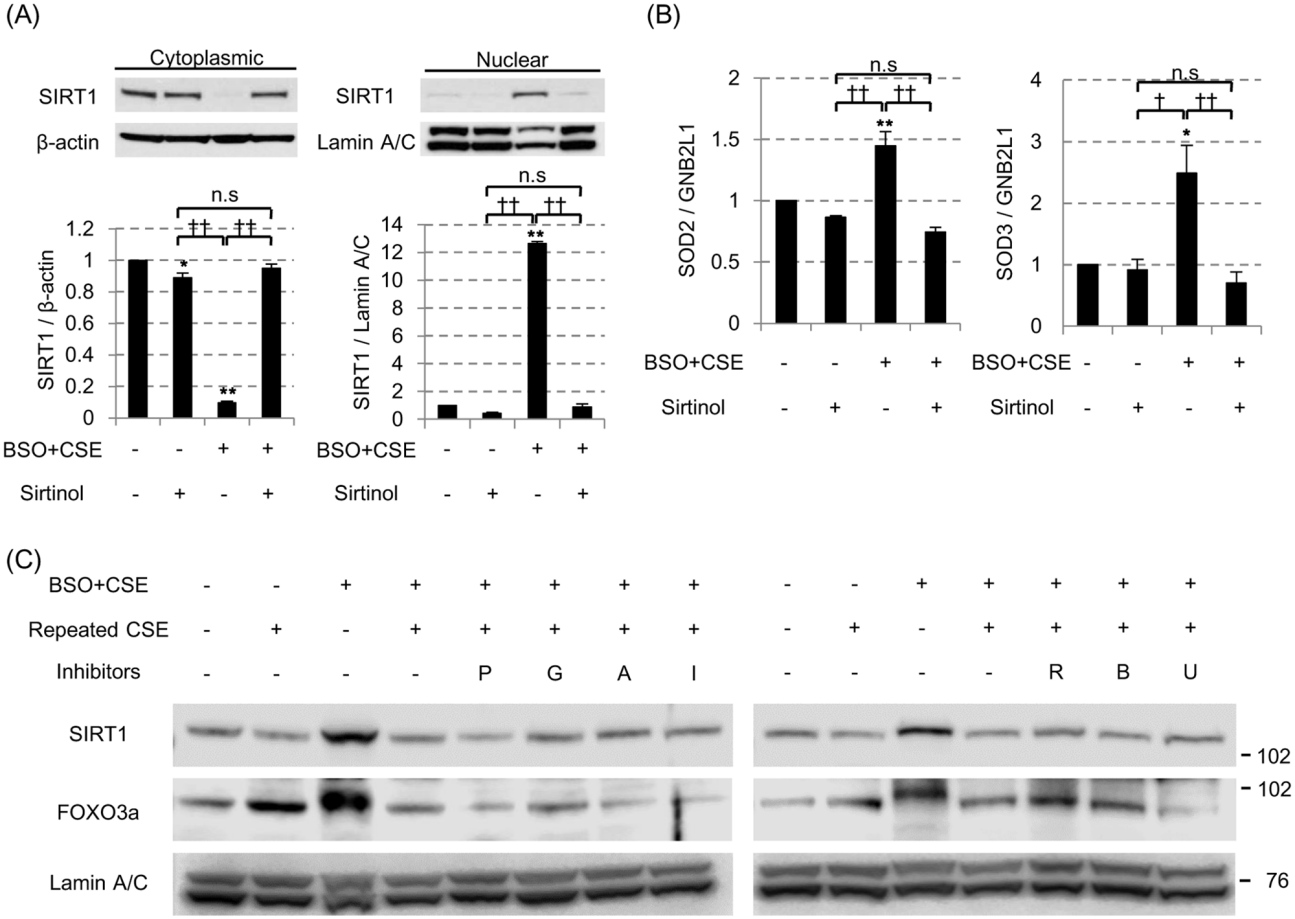
Shuttling of SIRT1 to nuclei was impaired by SIRT1 inhibitor. (A) In the 3% CSE exposure with 100 μM BSO pretreatment model, the effect of the SIRT1 inhibitor sirtinol (10 μg/ml) on SIRT1 shuttling and on the mRNA levels of SOD2 (B, left) and SOD3 (B, right) were examined. All values are mean values ± SEM of at least three experiments. * *p* < 0.05, ** *p* < 0.01, compared with the values of non-treatment group; ^†^ *p* < 0.05, ^††^ *p* < 0.01, compared between two groups. (C) The various kinase inhibitors were added 30 min prior to the final 3% CSE exposure (0.1 μM of PIK75 [P] for PI3Kα, 10 μM of GSK2636771 [G] for PI3Kβ, 10 μM of AS605240 [A] for PI3Kγ, 5 μM of IC87114 [I] for PI3Kδ, 0.02 μM of rapamycin [R] for mTOR, 0.1μM of BIRB786 [B] for p38MAPK and 0.1μM of U0126 [U] for ERK signaling. SIRT1 and FOXO3a protein levels in nuclei were evaluated by SDS-PAGE / WB.

Although, the molecular mechanism of impaired SIRT1 shuttling is not clear, the pretreatment with a SIRT1 inhibitor (sirtinol, 10 μg/ml) almost totally blocked the CSE-induced SIRT1 nuclear localization under the oxidative stress (Fig 6A), with resultant inhibition of SOD2 and SOD3 mRNA induction (Fig 6B) although SIRT1 activator, resveratrol did not affect SIRT1 nuclear translocation (S5 Fig). Interestingly, CRM1 is degraded by repeated cigarette smoke, which was restored by the MG-132 treatment, resulting to enable the export of SIRT1 into cytoplasm (S6 Fig). We also evaluate the effects of PI3K inhibitors, p38MAPK inhibitors or ERK inhibitors as well as an mTOR inhibitor, but none of these inhibitors reversed the impaired SIRT1 nuclear translation by repeated CSE exposure (Fig 6C). Thus, recurrent smoking priming seemed to inactivate SIRT1, preventing SIRT1 from to translocating to nucleus, although the underlying molecular mechanisms are still unclear.

## Discussion

In the current study, we have shown for the first time that the SIRT1 locates mainly in the cytoplasm in both immortalized and human primary bronchial epithelial cells under normal condition. Our data suggest that a “single” oxidative stress event dynamically translocates SIRT1 to the nucleus where SIRT1 could exert its anti-oxidative properties, such as SOD2 and SOD3 mRNA induction, leading to reduction of cellular oxidative stress. However, repeated CSE pre-exposure attenuated the SIRT1 shuttling and caused prolonged exposure of oxidative stress, suggesting that the impaired SIRT1 shuttling might be a key molecular mechanism contributing to the development of cigarette smoke (CS)-associated diseases such as chronic obstructive pulmonary disease (COPD) and lung cancer.

In chronic smokers, the level of available glutathione in the whole body is decreased [26,27], which is even more evident in sputum [33] and skeletal muscle [34]. To mimic this, cells were treated with BSO, which is known to deplete intracellular glutathione storage, making cells more susceptible to the exogenous oxidative stress [25]. In fact, BSO pretreatment augmented CSE-induced SIRT1 reduction; in that condition, as low as 3% CSE could effectively suppress the SIRT1. Importantly, the cell toxicity in this condition was milder than that in high CSE exposure without BSO; these results suggested that CSE exposure with BSO pretreatment might be more appropriate model for analyzing chronic effect of cigarette smoking.

According to our data, the activation of PI3K seem to be associated with the SIRT1 reduction under the oxidative stress, and we identified the PI3Kα isoform among 4 isoforms (p110α, p110β, p110γ and p110δ) [35] to be involved in SIRT1 shuttling in bronchial epithelial cells by pharmacological evaluation. PIK75, a potent PI3Kα inhibitor, is also known to inhibit DNA-PK. As shown in Fig 2D and 2E, an IC_50_ value of PIK75 on CSE-induced Akt phosphorylation was similar to the IC_50_ for PI3Kα enzyme inhibition as previously published [28], and also another DNA-PK inhibitor (NU7026) did not affect SIRT1 level. Therefore, the PI3Kα isoform is dominantly involved in SIRT1 reduction by CSE exposure. Previously, we had reported that PI3K signaling, especially the δ isoform is activated and up-regulated in the presence of oxidative stress, with this being associated with corticosteroid refractory inflammation especially in monocytic cells, macrophages or peripheral blood mononuclear cells (PBMCs) [36–39]. In the present study, no effect was seen of PI3Kδ inhibitor (IC87114) on the SIRT1 reduction under oxidative stress. This might be explained by the fact that the p110α is the most abundant isoform in normal bronchial epithelial cells and PI3Kδ isoform is expressed primarily in immune cells [40,41]. Interestingly, nicotine, an Akt activator, did not induce SIRT1 shuttling and Akt inhibitor did not inhibit SIRT1 shuttling stimulated by 3% CSE. Thus, SIRT1 nuclear translocation is regulated downstream of PI3Kα before Akt comes into play. PI3Kα is known to phosphorylate other proteins directly [42], and it might happen independently from Akt signaling. In addition, hydrogen peroxide is also reported to induce SIRT1 nuclear translocation in human kidney cells via JNK1 activation [30]. However, Anisomysine, JNK activator, did not induce SIRT1 shuttling, and also JNK inhibitor did not inhibited CSE-induced SIRT1 shuttling. Therefore, in human bronchial epithelial cells or cigarette smoke stimulation, JNK1 is unlikely involved in SIRT1 shuttling.

Although we found SIRT1 shuttling from cytoplasmic to nuclei by CSE stimulation, these findings were different from the previous report by *Caito et al* [43]; in which, the oxidative stress is reported to accelerate the nuclear to cytoplasmic export of SIRT1, leading to SIRT1 degradation promptly through the proteasomal pathway in BEAS-2B cells. However in this manuscript, SIRT1 was detected using immunofluorescent assay (IFA), which is sometimes unreliable method because of the incomplete epitope specificity that each antibody recognize, therefore it is not clear whether the antibody detected a full length protein, truncated protein or just fragments. Such controversy about the results of IFA were appropriately discussed in the report by *Byles et al* [24]; in that report, they finally concluded that intracellular SIRT1 location should be confirmed by the Western blotting (WB) combined with subcellular fractionation. We confirmed the SIRT1 location through the various methods such as 1) the WB combined with the subcellular fractionation, 2) the use of several anti-SIRT1 antibodies recognizing different SIRT1 epitopes, and 3) the use of immunofluorescent assay. We think our results to be more reliable and concluded that the SIRT1 locates predominantly in the cytoplasm under normal conditions.

The dynamic shuttling properties under oxidative stress seemed exclusively specific to SIRT1 compared with other de-acetylating enzymes such as HDAC2 or SIRT6. The reasons why SIRT1 shuttles between cytoplasm and nuclei in the presence of oxidative stress remains unclear. However, some reports suggested that cytoplasmic SIRT1 functions as a reserve, in case of an emergency [31]; it is therefore believed that under the condition such as oxidative stress exposure, cytoplasmic SIRT1 translocates to the nucleus, exerting its cellular anti-oxidative properties. In the presence of oxidative stress, SIRT1 is known to deacetylate FOXO3a [32], which then induce anti-oxidative proteins such as SOD2, SOD3, NQO-1 and HO-1 [32,44,45]. In fact, we confirmed FOXO3a nuclear translocation in CSE stimulated cells in the presence of BSO. Thus, the response of “single” CSE exposure is for adaptation and not appropriate *in vitro* model of CS-associated disease due to lack of chronic exposure of cigarette smoke.

Therefore, we next examined if the repeated CSE pre-exposure (rCSE) affected SIRT1 shuttling. For that objective, we co-stimulated BEAS-2B cells not only with lower concentration of CSE but also with low concentration of MG-132 (0.1 μM), so as to inhibit the proteasome degradation of SIRT1 and other proteins, which is reported in the cigarette smoke-associated diseases [46]. Although the repeated lower dose CSE (0.3%) pre-exposure itself did not have any effects on SIRT1 shuttling, the repeated 0.3% CSE exposure in the presence of MG-132 (0.1 μM) clearly impaired SIRT1 shuttling. Considering that MG-132 prevents the CRM1 degradation under CSE exposure, preserved CRM1 might contribute to the accelerated extra-nuclear export of SIRT1; this might be the one reason why MG-132 is necessary for the impaired SIRT1 shuttling on repeated CSE model. These rCSE priming seems to be the reasonable *in vitro* model for analyzing the impaired SIRT1 dynamics *in vivo*, because the primary bronchial epithelial cells from COPD subjects also showed similar dysregulation of SIRT1 shuttling.

The molecular mechanism behind impaired SIRT1 shuttling to nucleus by repeated pre-exposure of low dose CSE was still unclear. To understand this, we used multiplekinase inhibitors targeting PI3K, p38MAPK, ERK and mTOR, but all failed to reverse the impaired SIRT1 shuttling. We next investigated post-translational modifications of SIRT1, such as oxidation, phosphorylation, ubiquitination and acetylation, but we did not find any changes in the post-translational modifications of SIRT1 at the condition tested. However data did suggest that the sirtuin inhibitor, sirtinol, completely inhibited SIRT1 nuclear shuttling and resultant SOD2 and SOD3 induction under oxidative stress, mimicking repeated CSE exposure. Previous report [22] elucidated through a mutant study that; 1) SIRT1 has two nuclear export signals (NES1 and NES2), and 2) strong nuclear export activity is mainly attributed to the NES2, which locates inside the SIRT1 catalytic motif. Therefore, it might be possible that sirtinol not only binds to the catalytic domain to inhibit its enzyme activity but also modified the activity of NES2, which then impair the SIRT1 shuttling. Further studies are required to identify the molecular mechanism.

Our present study had some limitations. At first, SIRT1 shuttling would not be the only mechanisms for SIRT1 reduction under oxidative stress, despite its importance in CSE exposure (for up to 24 hours). Considering that the SIRT1 mRNA began to decrease at 24 hours, other mechanisms would also contribute to SIRT1 protein reduction at later phase; for example, SIRT1 mRNA instability by micro (mi)RNA would be the possible mechanism to be explored [47] as well as proteasome/autophagy systems. Secondary, although we considered SIRT1 shuttling to be cell-protective, these oxidant-resistant properties might contribute not only to cell survival but also to the malignant transformation of the cell, with resultant lung cancers as an important other CS-associated disease other than COPD. Such double-edged sword effects of SIRT1 shuttling might positively or negatively influence the patients’ prognosis [48]. Therefore, the balancing mechanisms between the cell protective/anti-oxidative properties and the resultant risk for malignant transformation might also be explored in the future study. We also performed preliminary staining of lung tissue on histological slides and could not detect any SIRT1 within the nucleus. To compare the level of SIRT1 shuttling in non-COPD and COPD, we would have to get fresh lung samples, and stimulate with CSE, or obtain fresh lung biopsies after CSE challenge in humans *in vivo*. Thus, further clinical study will be required.

In conclusion, cytoplasmic SIRT1 in bronchial epithelial cells seems to function as a reserve, and after oxidative stress exposure it translocates to the nucleus to exert its cellular anti-oxidative properties. We also have shown that impaired nuclear shuttling of SIRT1 might be associated with chronic CS exposure related diseases. Thus this impaired SIRT1 shuttling might be a possible therapeutic target for treating CS-associated diseases, and our findings also stress the need for smoking free environment.

## Supporting information

S1 Fig Cigarette smoke extract (CSE)-induced SIRT1 reduction. (A) BEAS-2B cells were incubated with various concentration of CSE for 24 hours, and SIRT1 protein levels in whole cell extract (WCE) were assayed by sodium dodecyl sulfate-polyacrylamide gel electrophoresis (SDS-PAGE) / Western blotting (WB). (B) Time-dependency of SIRT1 reduction in the presence of 20% CSE was examined at different time point up to 24 hours. ** *p* < 0.01, compared with the values of non-treatment group.
(TIF)

S2 Fig CSE-induced SIRT1 reduction was not due to proteasome degradation, reduction of transcription or translational dysregulation. (A) BEAS2B cells were treated with or without 3% CSE / 100 μM BSO, in the presence or absence of proteasome inhibitors such as Z-Leu-Leu-Leu-al (MG-132: 8 μM) or ALLN (N-acetyl-Leu-Leu-Norleu-al: 10 μM). After 24 hours exposure with CSE, SIRT1 protein levels were analysed. The Nrf2 was used as the control protein affected by proteasome inhibitors. (B) In the presence or absence of 3% CSE / 100 μM BSO, protein half-life were examined using cyclohexamide (CHX: 100 ng / ml) pretreatment. Then, the levels of SIRT1 were plotted at each time point, and protein half-life was investigated for each condition. (C) The translational regulation of SIRT1 was assayed using Click-IT^®^ labelling method. Newly synthesized SIRT1 were tagged with biotin, which were then immunoprecipitated and detected by SDS-PAGE/WB. The biotin-SIRT/Total-SIRT were used for assessing the nascent protein synthesis under the condition with or without BSO in the presence or absence of 3% CSE. (D) Samples were also collected 24 hours after CSE stimulaton, and SIRT1 mRNA level was examined by RT-qPCR. (E) Time-dependent changes of SIRT1 mRNA levels were also analyzed in the presence of PIK75 (0.1 μM). (F) Compare the ability of various antibodies that recognize different motif of SIRT1 to detect SIRT1 protein modified by oxidative stress. (G) SIRT1 secretion in the cell culture supernatants were assayed. All values are mean values ± SEM of at least three experiments. * *p* < 0.05, ** *p* < 0.01, compared with the values of non-treatment group; ^††^ *p* < 0.01, compared with or without 0.1 μM PIK75; NLS = nuclear localization sequence; NES = nuclear export sequence.
(TIF)

S3 Fig Full image of Western blotting of SIRT1 and housekeeping genes shown in Fig 3A and 3B. (A) Cytoplasmic fraction was analyzed for SIRT1 (upper) and β-actin (lower). (B) Nuclear fraction was analyzed for SIRT1 (upper) and Lamin A/C (lower).
(TIF)

S4 Fig Time-dependency of Cigarette smoke extract (CSE)-induced expression of SIRT1 in nuclear fraction in the presence of BSO with/without priming with repeated 0.3% CSE. BEAS2B cells were treated with or without 3% CSE / 100 μM BSO for 4, 8, 16 and 24 hours or treated with 0.3% CSE in the presence of MG123 for 3 days and then stimulated with 3% CSE in the presence of 100 μM BSO for 4, 8, 16 and 24 hours. SIRT1 protein levels in cytoplasm (A) and nuclei (B) were assayed by sodium dodecyl sulfate-polyacrylamide gel electrophoresis (SDS-PAGE) / Western blotting (WB). (C) Malondialdehyde (MDA) was measured in cytoplasm fraction (n = 3).
(TIF)

S5 Fig Effects of resveratrol on CSE-induced SIRT1 nuclear translocation. (A) BEAS2B cells were treated with Resveratrol (10μM) in the presence of BSO and stimulated with 3%CSE.
(TIF)

S6 Fig Effects of MG132 on SIRT1, FOXO3a, CRM1 and Lamin A/C protein expression in nuclei after repeated 0.3% CSE exposure. MG-132 was treated before every CSE treatment.
(TIF)
