# Supplementary figures and images for: The dynamic shuttling of SIRT1 between cytoplasm and nuclei in bronchial epithelial cells by single and repeated cigarette smoke exposure
Source: PLoS One. 2018 Mar 6;13(3):e0193921. doi: 10.1371/journal.pone.0193921 (PMC5839577; doi:10.1371/journal.pone.0193921)

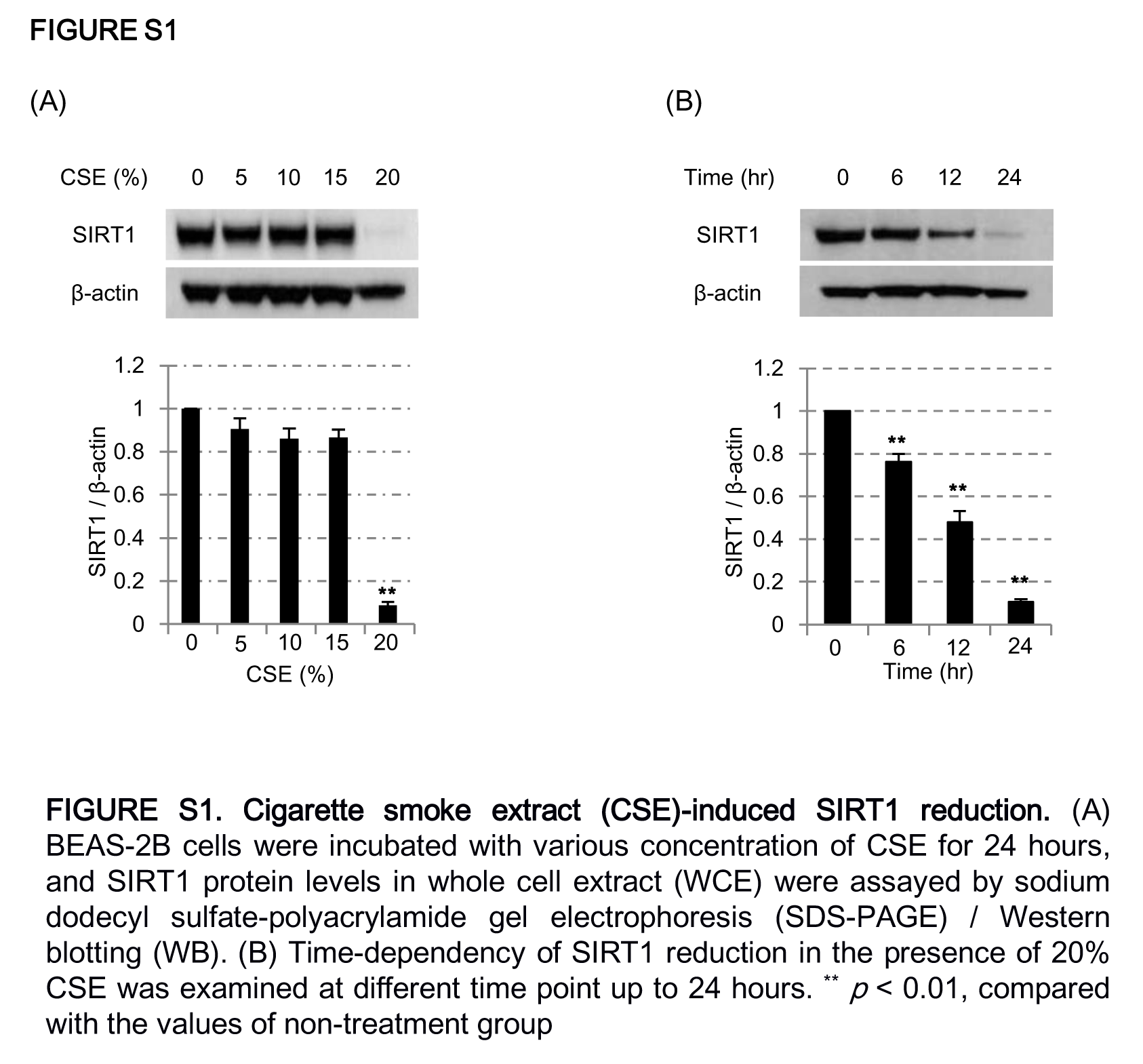

Supplement: S1 Fig — (A) BEAS-2B cells were incubated with various concentration of CSE for 24 hours, and SIRT1 protein levels in whole cell extract (WCE) were assayed by sodium dodecyl sulfate-polyacrylamide gel electrophoresis (SDS-PAGE) / Western blotting (WB). (B) Time-dependency of SIRT1 reduction in the presence of 20% CSE was examined at different time point up to 24 hours. ** p < 0.01, compared with the values of non-treatment group. (TIF) [file pone.0193921.s001.tif]

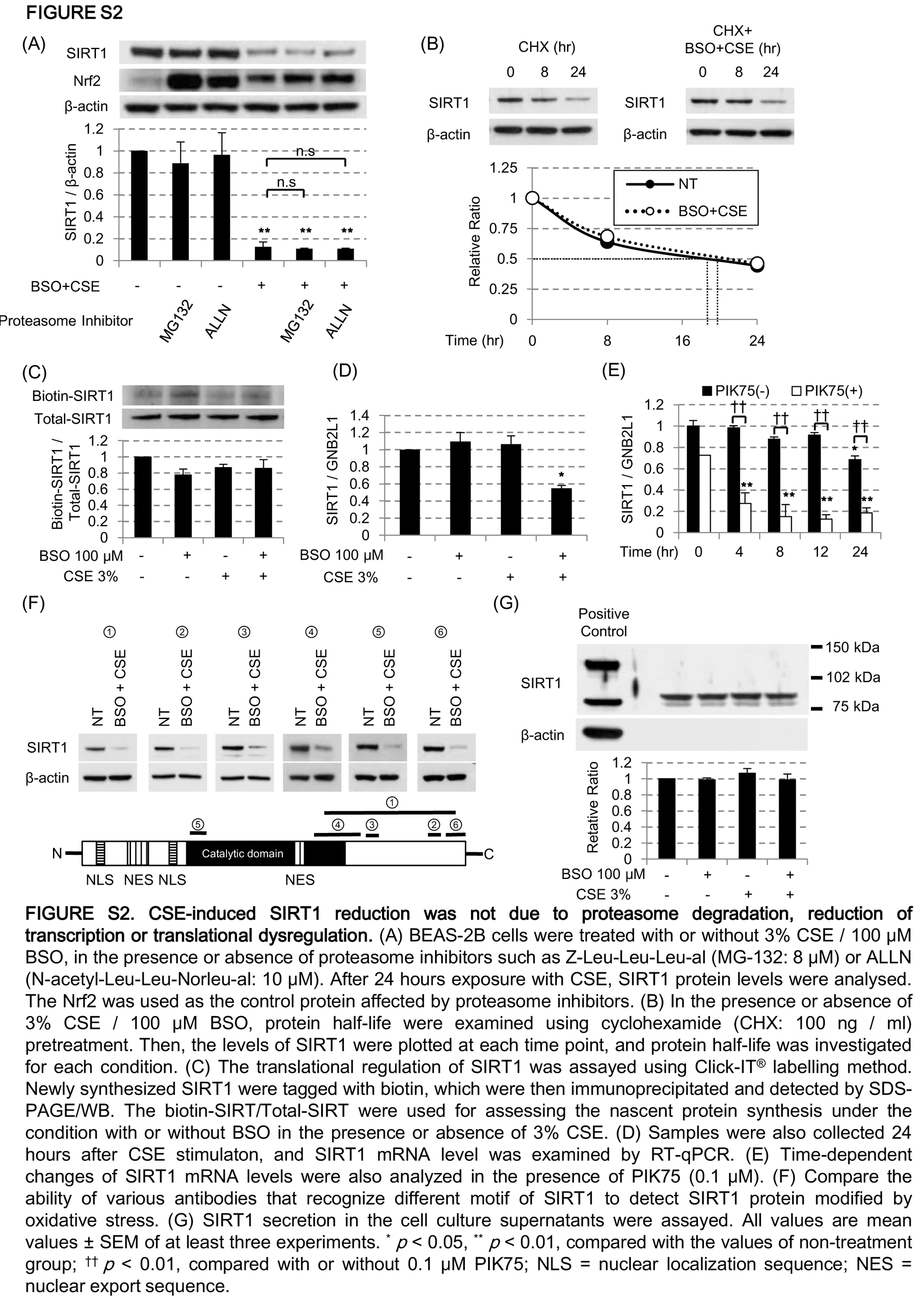

Supplement: S2 Fig — (A) BEAS2B cells were treated with or without 3% CSE / 100 μM BSO, in the presence or absence of proteasome inhibitors such as Z-Leu-Leu-Leu-al (MG-132: 8 μM) or ALLN (N-acetyl-Leu-Leu-Norleu-al: 10 μM). After 24 hours exposure with CSE, SIRT1 protein levels were analysed. The Nrf2 was used as the control protein affected by proteasome inhibitors. (B) In the presence or absence of 3% CSE / 100 μM BSO, protein half-life were examined using cyclohexamide (CHX: 100 ng / ml) pretreatment. Then, the levels of SIRT1 were plotted at each time point, and protein half-life was investigated for each condition. (C) The translational regulation of SIRT1 was assayed using Click-IT® labelling method. Newly synthesized SIRT1 were tagged with biotin, which were then immunoprecipitated and detected by SDS-PAGE/WB. The biotin-SIRT/Total-SIRT were used for assessing the nascent protein synthesis under the condition with or without BSO in the presence or absence of 3% CSE. (D) Samples were also collected 24 hours after CSE stimulaton, and SIRT1 mRNA level was examined by RT-qPCR. (E) Time-dependent changes of SIRT1 mRNA levels were also analyzed in the presence of PIK75 (0.1 μM). (F) Compare the ability of various antibodies that recognize different motif of SIRT1 to detect SIRT1 protein modified by oxidative stress. (G) SIRT1 secretion in the cell culture supernatants were assayed. All values are mean values ± SEM of at least three experiments. * p < 0.05, ** p < 0.01, compared with the values of non-treatment group; †† p < 0.01, compared with or without 0.1 μM PIK75; NLS = nuclear localization sequence; NES = nuclear export sequence. (TIF) [file pone.0193921.s002.tif]

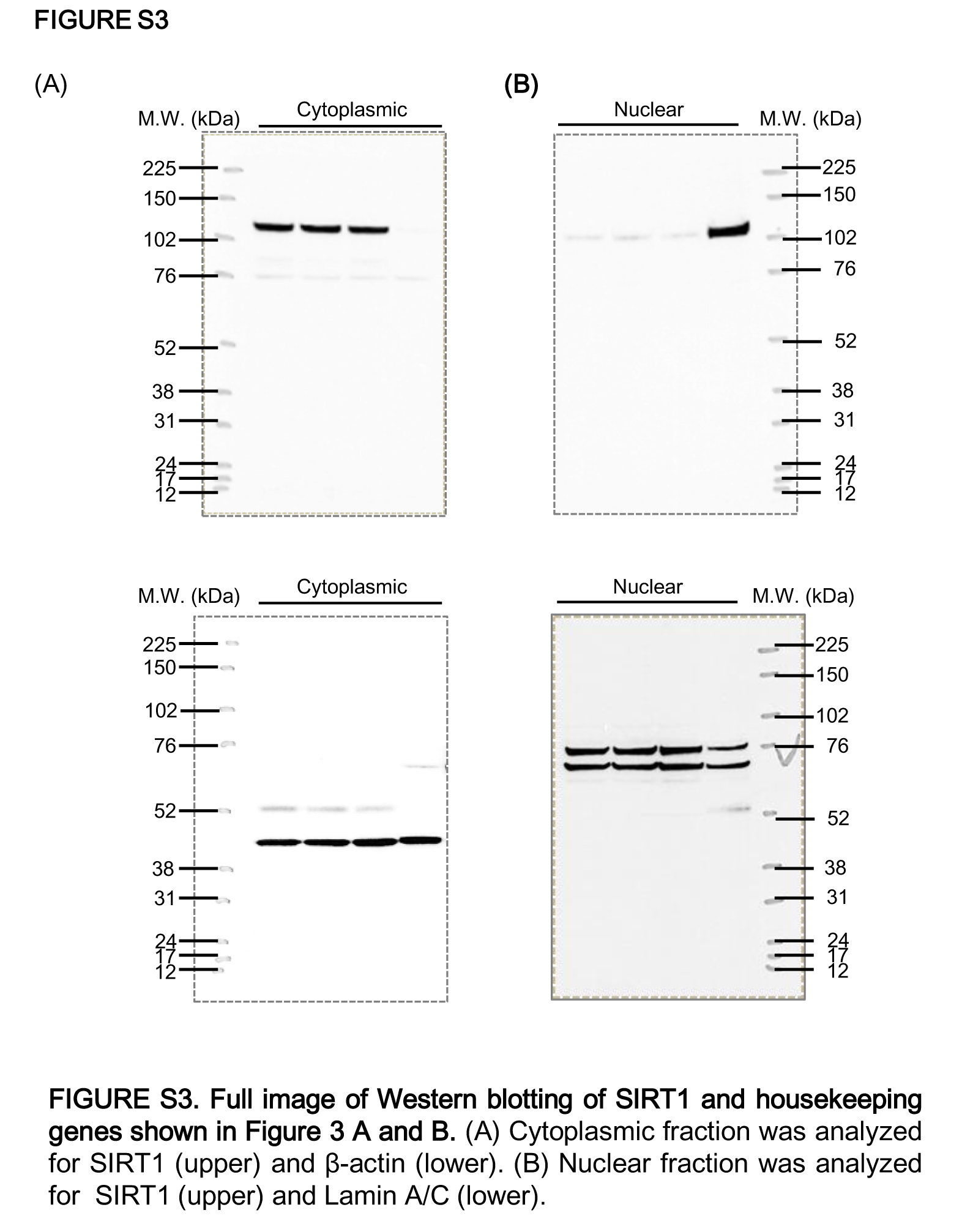

Supplement: S3 Fig — (A) Cytoplasmic fraction was analyzed for SIRT1 (upper) and β-actin (lower). (B) Nuclear fraction was analyzed for SIRT1 (upper) and Lamin A/C (lower). (TIF) [file pone.0193921.s003.tif]

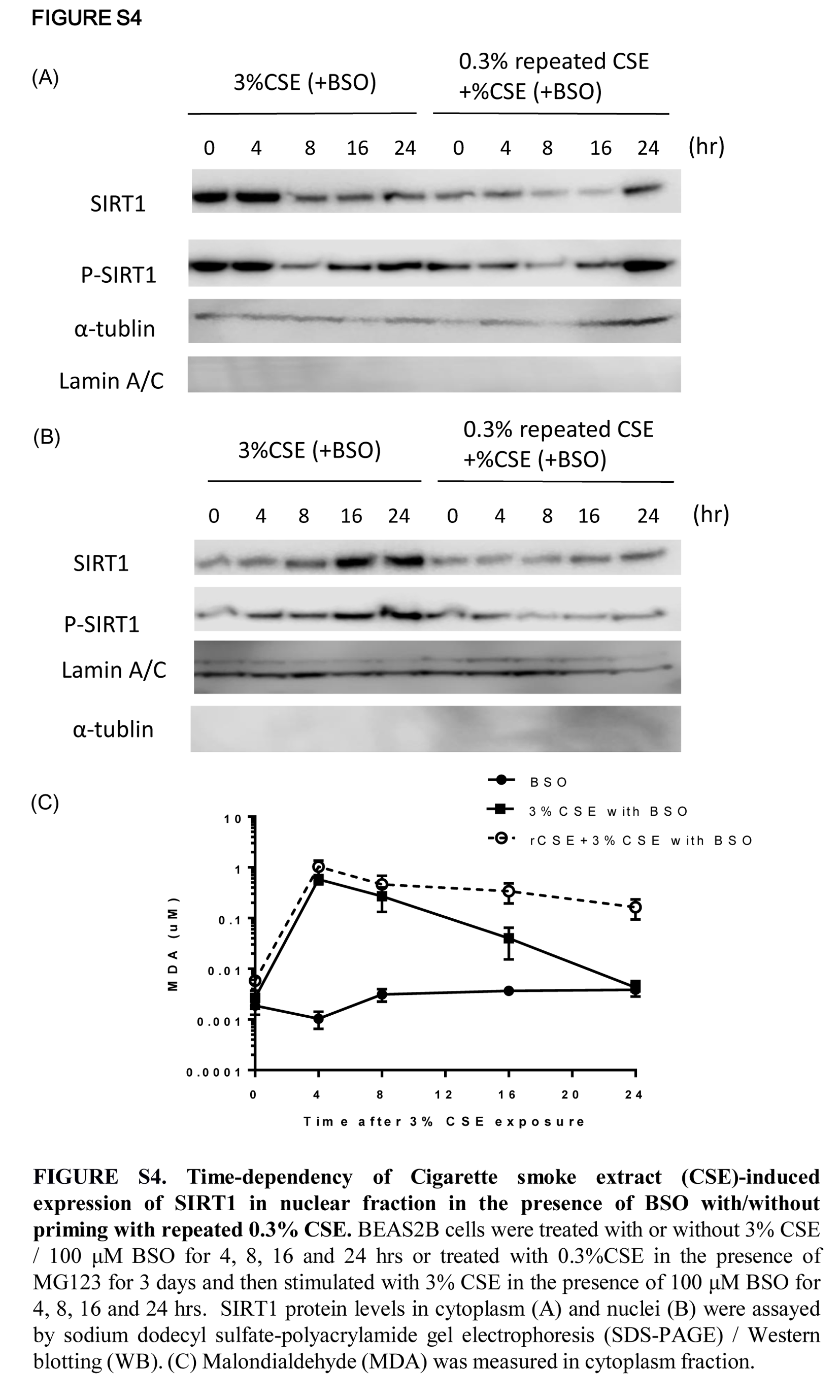

Supplement: S4 Fig — BEAS2B cells were treated with or without 3% CSE / 100 μM BSO for 4, 8, 16 and 24 hours or treated with 0.3% CSE in the presence of MG123 for 3 days and then stimulated with 3% CSE in the presence of 100 μM BSO for 4, 8, 16 and 24 hours. SIRT1 protein levels in cytoplasm (A) and nuclei (B) were assayed by sodium dodecyl sulfate-polyacrylamide gel electrophoresis (SDS-PAGE) / Western blotting (WB). (C) Malondialdehyde (MDA) was measured in cytoplasm fraction (n = 3). (TIF) [file pone.0193921.s004.tif]

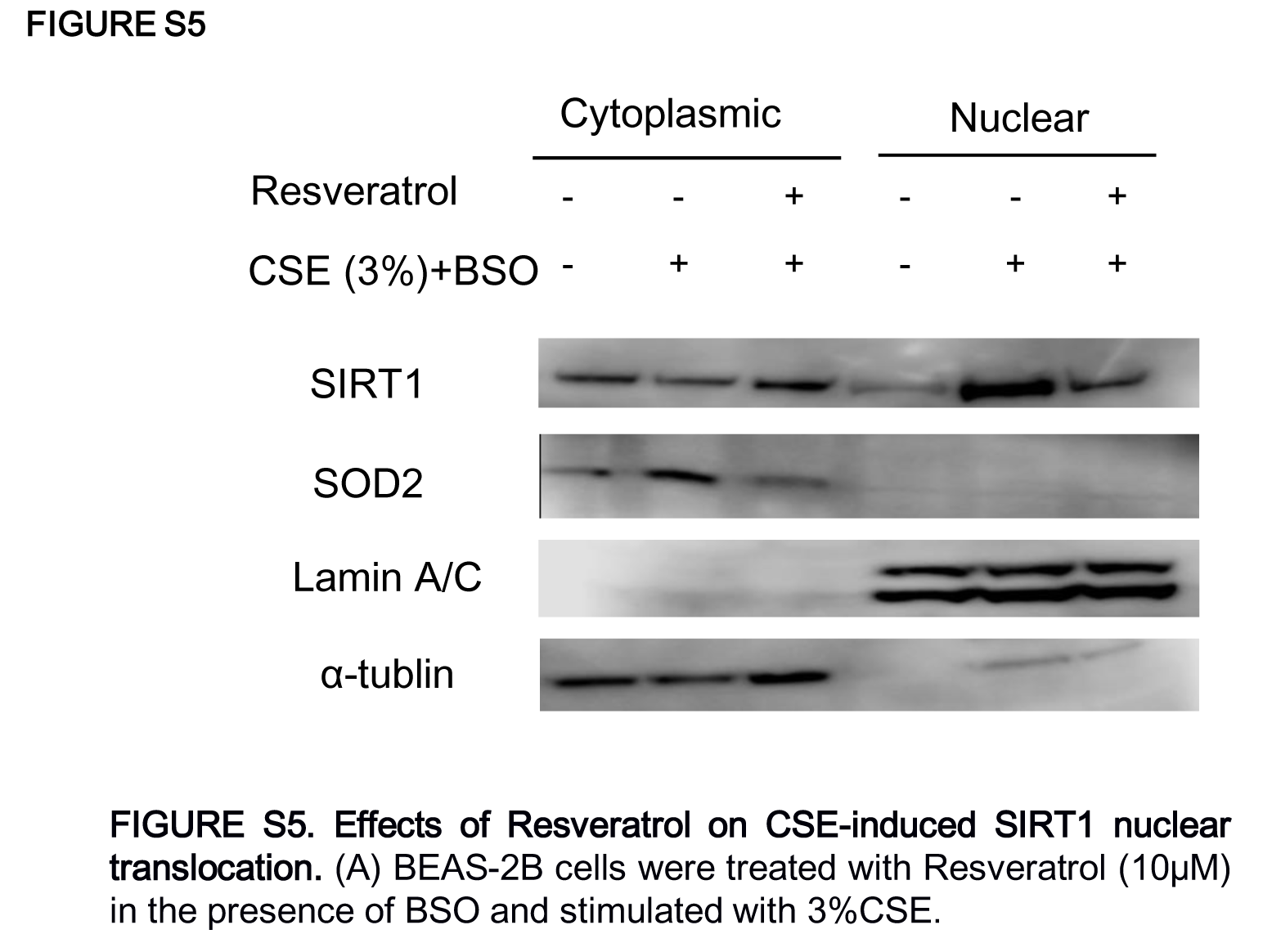

Supplement: S5 Fig — (A) BEAS2B cells were treated with Resveratrol (10μM) in the presence of BSO and stimulated with 3%CSE. (TIF) [file pone.0193921.s005.tif]

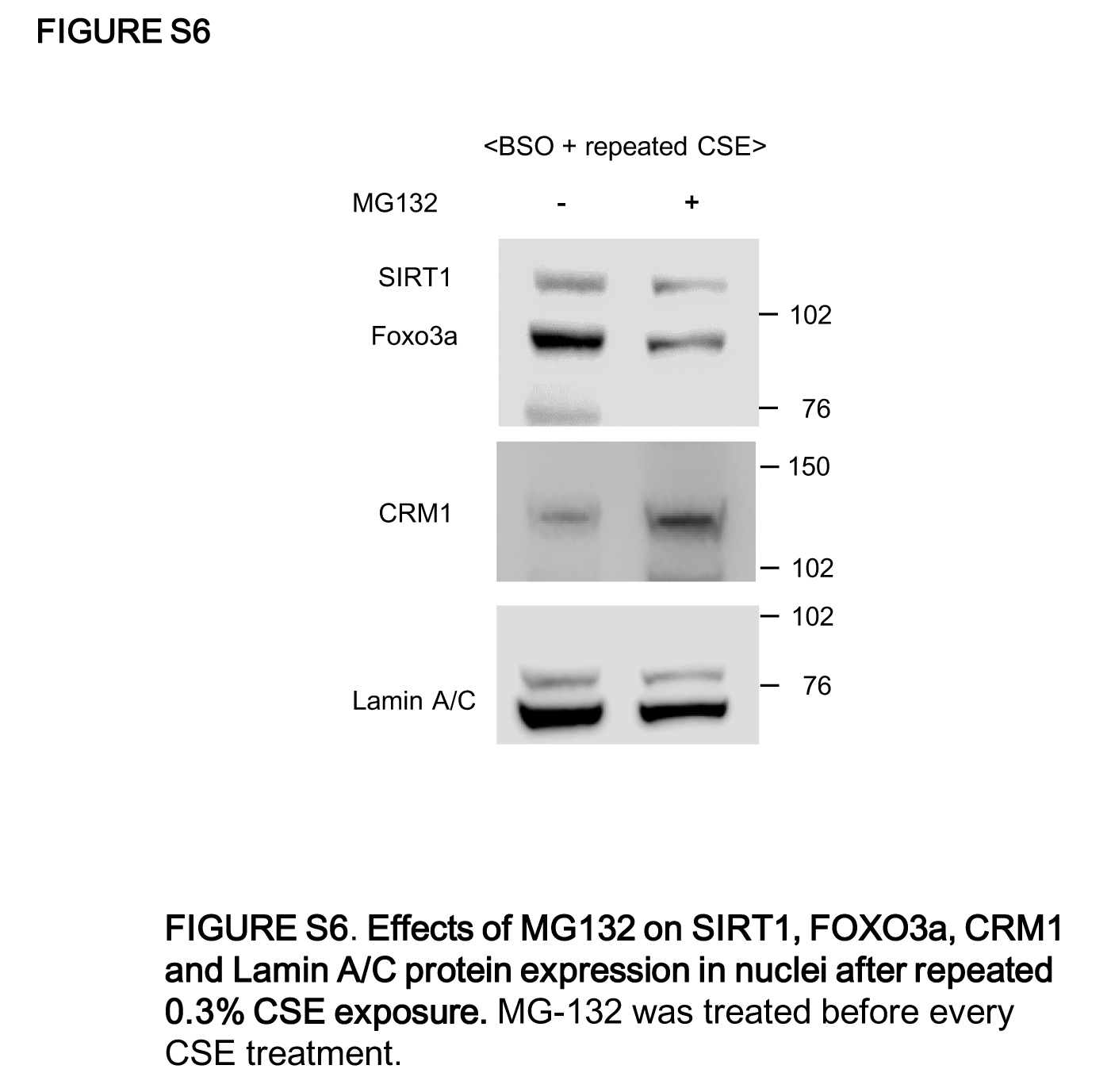

Supplement: S6 Fig — MG-132 was treated before every CSE treatment. (TIF) [file pone.0193921.s006.tif]
